# Risky sexual behavior and associated factors among sexually-experienced adolescents in Bangkok, Thailand: findings from a school web-based survey

**DOI:** 10.1186/s12978-022-01429-3

**Published:** 2022-05-28

**Authors:** Bang-on Thepthien, Celyn

**Affiliations:** 1 ASEAN Institute for Health Development, Mahidol University, 25/25, Phutthamonthon 4 Rd, Nakhon Pathom, 73170 Thailand; 2 Taunggyi University, Taunggyi, Shan State Myanmar

**Keywords:** Risky sexual behavior, Sexually-experienced, Adolescents

## Abstract

**Background:**

The risk of sexually transmitted infections (STI) arises when there is unsafe sexual activity. Unsafe sex often begins in the teenage years, and it will persist as long as there is the opportunity for risky sexual activity. The purpose of this study was to assess the sexual risk behaviors and related factors of sexually-active adolescents in educational institutions in Thailand.

**Methods:**

This was cross-sectional survey using an Internet-based application in schools in Bangkok from November 2020 to February 2021 with a total of 6,167 high school and vocational students. The schools were selected by simple random sampling among institutions, and the students were systematically randomly selected, with an equal number of males and females. We used multivariable logistic regression to analyze associations and control confounding variables. Indicators of risky sex include: (1) Not using a condom; (2) Having more than one sex partner; (3) Having sex in exchange for cash or in-kind compensation; and (4) Having sex without consent.

**Results:**

Of a total of 872 sexually-active participants, the mean age was 15.6 years, 66.9% were vocational students, 42.1% were male, 57.9% were female, and 69.5% had sex risk behavior. The multivariate logistic regression analysis identified the following statistically-significant factors related to risky sex: smokes cigarettes (AOR = 1.79; 95% CI 1.12–2.88); uses cannabis (AOR = 2.84; 95% CI 1.23–6.56); gambles (AOR = 1.81; 95% CI 1.28–2.55); has sex without contraception (AOR = 2.74; 95% CI 2.91–3.93); has a history of childhood sex abuse (AOR = 1.60; 95% CI 1.03–2.56).

**Conclusions:**

Our findings suggest that, in designing and implementing sexual risk prevention programs for adolescents in educational settings, there is a need to highlight the role of substance abuse in relation to sexual risk behaviors. Programs should target both male and female students. Teenagers who use marijuana are more likely to be sexually active than those who have never used marijuana. These potential risks should be taken into account with respect to legalization of marijuana and recreational use of this drug.

## Plain Language Summary

Risky sexual behavior (RSB) is engaging in sexual practices that may increase vulnerability to a reproductive health problem. This study was conducted to assess the sexual risk behaviors of students living in Bangkok, which has the highest rates of STIs among regions in Thailand. Previous studies of RSB have focused on the general population age 18 years or older. By contrast, there is a dearth of studies on sex behavior among Thais under age 18 years. In addition, the present study aimed to explore the association of substance abuse and adverse childhood experiences (ACEs) with adolescent RSB. In this study, 14.1% of all students in the participating schools had ever had sex before. Using the study criteria, two out of three students in the sample had experienced RSB. The findings from this study may help inform programs and interventions aimed at reducing the negative health impacts associated with RSB, specifically STIs. The findings of the study should be relevant for health program managers, socio-behavioral researchers, and other stakeholders who design reproductive health intervention strategies for in-school adolescents.

## Introduction

In the world today, there are about 1.2 billion persons between the ages of 10 and 19 years, or 16% of the total global population. Over half of this age group lives in Asia. South Asia has nearly 350 million persons age 10–19 years, which is the most of any geographic sub-region. Next most populous is East Asia and the Pacific with over 300 million persons age 10–19 years [1]. Of the 66 million Thais, it is estimated that about seven million are age 10–19 years, or 10.7%. Of Bangkok’s 5.5 million registered population, about 600,000 are age 10 to 19 years, or 10.6% [2]. There is growing concern in Thailand about risky sex behavior (RSB), sexually transmitted infections (STI), and unplanned pregnancy among teenagers. Thus, studies are needed to define the scope of the problem and the factors associated with RSB among adolescents [3, 4]. The concept of safe sex needs to be promoted as a norm before adolescents become sexually active. Otherwise, it will be more difficult to change RSB once an adolescent acquires unhealthy habits. Adverse consequences of unsafe sex in adolescence include both harmful physical and psychosocial effects [5, 6]. In order to prevent the most harmful consequences of unsafe sex (e.g., HIV, incurable STIs, unplanned pregnancy, etc.) it is imperative to promote knowledge about responsible sex behavior before Thai youth become sexually active Thus, research into the sexual experience of today’s adolescents can help inform the education and prevention programs of tomorrow.

Cultural values and public health policies related to sexual behavior vary from country to country. In addition, cultural values and health policies have changed over time. As a result, gender norms in different parts of the world are experiencing cultural shifts, including Thailand [7]. The risk of the killer virus HIV from unsafe sex has been known for 40 years. Still, there are continuous reports of RSB with each new generation. Today’s adolescents have their sexual debut at an increasingly younger age, tend to have sex with multiple casual partners, prefer to use mood-altering drugs before and during sex, and are not conscientious about using effective contraception (especially condoms) [8]. Data for 2020 indicates that STIs are increasing in many age groups in Thailand, especially among adolescents, with an increase in morbidity from 79 per 100,000 population in 2010 to 191 per 100,000 population ten years later, or more than doubling [9]. This trend is a sign that the reproductive health of today’s generation of adolescents is under threat. At present, comprehensive sex education for pre-pubescent youth is woefully inadequate. One of the more serious indicators of this failing is that the leading cause of death for females age 15–19 worldwide is complications of pregnancy and childbirth.

Significant evidence suggests that physical abuse and child sexual abuse contribute to RSB in adolescents [10]. Adolescents who were sexually abused in childhood are at higher risk of premature sexual debut, having multiple sex partners, being a victim of coercive sex, engaging in casual sex, and having unprotected sex. Child physical abuse was also associated with a higher risk of sexual behavior at an older age, such as having sex with a casual partner or acquaintance, impulsive sexual behavior, and harmful anal sex in adolescence and young adulthood. Identifying the associations between predisposing factors and RSB is essential for the development of effective prevention and intervention strategies to address adolescent reproductive health, and prevent negative outcomes, particularly among at-risk populations. According to the literature, there is a link between physical abuse, child sexual abuse, and RSB in adolescence or young adulthood. However, the mechanisms underlying these connections is unclear. RSB may be used as a means of gaining love and reward after sexual abuse or to alleviate negative emotions caused by childhood abuse. Similarly, risk of substance abuse can increase when teens have difficulty controlling their behavior or when trying to reduce the internal turmoil of adolescence. According to the theory of self-healing, the self-medication theory suggests that a person may abuse substances (e.g., alcohol and other mood-altering drugs) to cope and compensate for the emotional disturbances associated with the harm sustained earlier in life. Substance abuse may lead to an increased risk of addictive sexual behavior by reducing inhibitions. According to myopia theory, alcohol may increase the likelihood of engaging in RSB by limiting perceptions and attention. Because of this, people who are intoxicated and have reduced impulse control, disproportionately focus on the incentives (e.g., arousal, lust) rather than the adverse consequences (e.g., STIs) of sex.

Substance abuse during adolescence interferes with normal cognitive, emotional, and social development. Several studies show that early and frequent substance abuse or increased substance abuse over time threaten development in adulthood, including attainment of educational goals and healthy physical and mental development [11]. A number of studies have examined the relationship between substance abuse and RSB, using cross-sectional or longitudinal data covering short periods of time [12]. In general, many of these studies have found a positive association between substance use and RSB. For example, some studies found that excessive alcohol use is positively associated with a greater number of sexual partners, and has a very negative effect on condom use [12–15]. Other studies have found that smoking is strongly associated with higher-risk sexual activity, including having a higher number of sex partners and lack of condom use [16]. The use of marijuana and other mood-altering drugs is associated with having a higher number of sexual partners and lack of condom use [17]. On the other hand, some studies have not found a significant relationship between use of alcohol, marijuana, tobacco, and/or cocaine and condom use [18, 19].

In Thailand, secondary school is usually a new environment for the young adolescent, in which people from different primary schools come together and are exposed to a much wider range of attitudes and behaviors of their student peers, including sexual norms and behavior. The vast majority of students attending (general) secondary schools are in their teens, where gender identity formation and gender experimentation take place. Students who are tracked into vocational education usually continue toward a certificate/diploma or commercial college education. By contrast, students in general high schools are exposed to a range of courses and disciplines, usually with the goal of preparing them to pass the college entrance exams for study toward earning a bachelor’s degree or higher. Of course, high school and vocational school is a period that coincides with transitioning through puberty, hormonal changes of adolescence, and the experience of powerful drive to explore issues about sex and experiment with sexual behavior [20]. This is a universal phenomenon of adolescents in all societies around the world—unless the state or culture imposes strict restrictions on adolescent attire and unsupervised interaction between the sexes [21].

The objective of this study was to assess the RSB of students living in Bangkok, which has the highest rates of STIs among regions in Thailand. Previous studies of RSB have focused on the general population age 18 years or older. By contrast, there is a dearth of studies on sex behavior among Thais age under 18 years. In addition, the present study aimed to explore the association of substance abuse and adverse childhood experiences (ACEs) with adolescent RSB. Past research has found a statistically significant association between history of ACEs and RSB in adolescence. However, most of those studies were conducted in Western countries; very few have been conducted in Thailand. The findings from this study should help inform programs and interventions aimed at reducing the negative health impacts associated with RSB, specifically STIs. The findings of the study should be relevant for health program managers, socio-behavioral researchers, and other stakeholders who design reproductive health intervention strategies for in-school adolescents.

## Materials and methods

### Study area and period

Data were collected for this study as part of a Bangkok Behavioral Surveillance Survey (BBSS) project, which monitors HIV infection and risk factors in ten target populations in the Bangkok metropolitan area. The BBSS is conducted every two years, and includes a sample of students enrolled in high school Years 2 and 5, and vocational Year 2 students from 26 high schools and 15 vocational schools in Bangkok. The cross-sectional data collection was conducted in February 2020 by applying an Internet-based survey using Lime software. The format was optimized for both computers, tablets, and mobile devices. The questionnaire took an average of 15 min to complete.

### Population and eligibility

The registered population in Bangkok at the time of the survey was 5,588,222 people, with persons age 10–19 years totaling 591,596 people, and persons age 14–16 years totaling 59,139. ​​Data were collected from 6167 students representing about 10% of adolescents in educational institutions in Bangkok at the time. A total of 872 respondents reported that they had ever had sex before, or 14.1% of all adolescents included in the BBSS. Among the 872 sexually-active students, 66 were studying in high school Year 2, 223 were in high school Year 5, and 583 were in their 2nd year of vocational school.

### Sample size and sampling technique

The schools were selected by simple random sampling among institutions with at least 1000 students enrolled, and stratified by six geographic zones of Bangkok. In the final step, 100–120 students in each school were systematically, randomly selected, with an equal number of males and females.

### Data collection instruments and procedures

The Sexual Behavior Module was developed from the National Youth Risk Behavior Survey questionnaire. The independent variables used in the study include socio-demographic factors (gender, GPA in the last semester, migration status, location of parents’/guardians’ home, other members of the current residence, cost of living, monthly allowance, grade level). Personal behavior variables include alcohol consumption, smoking, vaping, marijuana use, pornography viewing, gambling, ACEs, pregnancy, and contraception. Peer-related factors include having close friends involved in substance abuse, and having close friends who tried to persuade the respondent to use drugs. School-related variables include perception of strict sanctions against student substance abuse and safety. Family factors include whether the parents are divorced, and presence of domestic violence. Respondents were asked if they used contraception at last sex. If the response was “yes,” they were asked if they used emergency contraception or some other form of post-coital prevention of pregnancy.

The Substance Abuse Module asked respondents about their use of alcohol, tobacco cigarettes, and e-cigarettes (i.e., vaping) in the past month. Respondents were asked about cannabis use in the past year. Alcohol consumption was measured by asking the following question: “*During the past month, did you drink one or more servings of an alcoholic beverage at one sitting?”* (“Beverage” refers to a glass of wine, a bottle of wine cooler, a small bottle/can of beer, a shot of liquor, or a mixed drink.) Smoking (tobacco and or vaping) was measured by the question: “*Did you smoke at least once in the past month?”* Marijuana use was measured by response to the question: “*Did you smoke marijuana at least once in the last year?”.*

After receiving approval from the Ethical Review Committee, the researchers asked the participating schools to obtain consent from parents of students under 18 years of age to participate in the study. Student privacy was protected through anonymous and voluntary participation, and informed consent was obtained as appropriate from the students, parents and/or school officials. Students were assured that they were free to participate in the survey or not, that their responses would be totally confidential, and their choice/refusal to participate would have no effect on their student performance evaluation.

### Measurements and operational definitions

Risky sexual behavior (RSB) was the primary outcome variable. RSB was measured using a composite of the following indicators: (1) Having sex with a commercial sex worker or sex in exchange for cash or in-kind compensation; (2) Having two or more sexual partners; (3–4) Practicing inconsistent condom use or failure to use condoms during intercourse at first and latest sex episode; and (5–6) Experiencing coercion at first sex; experienced coercive sex in the past year. Response was scored, with a total possible score = 6 points. If respondents answered '*Yes*' to a particular type of sexual behavior, it was defined as risky sexual behavior. In this study, RSB was calculated only among those students who had had at least one episode of sexual intercourse up to the time of data collection.

### Data processing and analysis

The questionnaire was initially checked with the Lime Survey software for completeness and consistency of response prior to the analysis. Data were then exported to SPSS version 25 for further analysis. The researchers conducted descriptive statistics, such as calculating frequency and percentages for discrete independent variables, and the mean (with standard deviation) for continuous variables. A binary logistic regression analysis was conducted to test for statistically-significant associations between the independent and dependent variables, with confidence level of p < 0.05 (95%), and remaining variables were introduced into the multivariate models. Variables were tested for multi-collinearity using the methodology proposed by Stevens (2012) [22]. The resulting *r* values were ​​not greater than 0.80, and model suitability was examined with the Hosmer-Leme test (P = 0.099) with a statistically significant level of p-value < 0.05.

## Study results

### Socio-demographic characteristics

In the study, 14.1% (95% CI: 13.3, 15.0) of all students in the participating schools had ever had sex before, including 7.6% of high school Year 2 students, 25.6% of high school Year 5 students, and 66.9% of vocational Year 2 students. The mean age of the study sample was 15.7 years, two-thirds (65.1%) were age above 15 years. Of the sexually active, two out of five (42.1%) were male students, while 57.9% were female students.

Of the 872 students who had ever had sex, more were female than male, and were more likely to be vocational students, followed by students in high school Years 5 and 2. Most of the respondents said they were living with their father and/or mother, and were born in Bangkok. About 60 percent lived in the family-owned dwelling. More than half the students perceived that their school was very strict about the prohibition of substance abuse. Half the students had low academic achievement. About one in four lived in a household with divorced parents and/or domestic abuse. Also, about one in four said a close friend tried to persuade them to use addictive drugs, and more male students reported that experience than their female counterparts. For details of the demographic, see Table 1.

**Table 1:** Socio-demographic characteristics of sexually-active adolescents in Bangkok, 2020 (n = 872)

Variables	Categories	Total (n = 872)		Sex category				P-Value ^a^
				Male (n = 367)		Female (n = 505)		
		n	%	n	%	n	%	
Living arrangements	With biological parents	610	70.0	260	70.8	350	69.3	0.625
	Away from parents^b^	262	30.0	107	29.2	155	30.7	
Residence	Own	519	59.5	235	64.0	284	56.2	0.006*
	Rent	312	35.8	110	30.0	202	40.0	
	Other	41	4.7	22	6.0	19	3.8	
Born in Bangkok	Yes	751	86.7	308	83.9	448	88.7	0.040*
	No	166	13.3	59	16.1	57	11.3	
Current grade level	High school Year 2	66	7.6	42	11.4	24	4.8	0.001**
	High school Year 5	223	25.6	96	26.2	127	25.1	
	Vocational Year 2	583	66.9	229	62.4	354	70.1	
Age (years) Mean = 15.7 SD = 1.6	13–15	2150	34.9	1141	39.8	1009	30.6	< 0.001
	16–19	4017	65.1	1728	60.2	2289	69.4	
Status	Live together	98	11.2	38	10.4	60	11.9	0.481
	No	774	88.8	329	89.6	445	88.1	
GPA	1–2	475	54.5	225	61.3	250	49.5	0.001**
	3–4	397	45.5	142	38.7	255	50.5	
Allowance (baht per day)^c^	≤ 100	237	27.2	105	28.6	132	26.1	0.64
	101–200	520	59.6	204	55.6	316	62.6	
	> 200	115	13.2	58	15.8	57	11.3	
A close friend tried to persuade them to use drugs	No	644	73.9	257	70.0	387	76.6	0.028*
	Yes	228	26.1	110	30.0	118	23.4	
Perception of school strictness about drugs	Low	410	47.0	187	51.0	223	44.2	0.047*
	High	462	53.0	180	49.0	282	55.8	
Parents are divorced	No	647	74.2	282	76.8	365	72.3	0.129
	Yes	225	25.8	85	23.2	140	27.7	
Domestic violence	No	670	76.8	291	79.3	379	75.0	0.143
	Yes	202	23.2	76	20.7	126	25.0	

^a^Chi-square test, ^b^living with relatives, living alone, living in group. ^c^1 USD = 33 baht

*p-value < 0.05, **p-value < 0.001

### Factors related to ACEs and substance abuse

As show in Table 2, Over two-thirds of the students (69.2%) had viewed pornographic media before, with males having a significantly greater proportion of porn-viewing behavior than females. Nearly one in seven (13.6%) of the sample had experienced sexual harassment, while over one in five (22.6%) had experienced physical and/or emotional abuse, and/or physical or emotional neglect (22.7%). About one-third have experienced substantial property loss from gambling. Males accounted for significantly more losses from gambling than females. About one in ten had a history of unintended pregnancy; 6.5% of male students reported that their partner had an unintended pregnancy, while 14.3 percent of female students reported that they had experienced an unintended pregnancy. Use of drugs included drinking at least one alcoholic beverage (40.9%), smoking tobacco (21.4%), and smoking e-cigarettes (20.8%) in the last 30 days. Recreational use of marijuana in the past year was reported by 6.9%, with males having a significantly higher proportion of substance use than females for most substances. The exception is for alcohol consumption, for which females reported significantly higher use than males. Over two-thirds of the respondents (68.8%) used contraception, and 7.9% had used emergency contraception. Fully, 7.8% reported that their first sex was involuntarily, and females had a significantly higher proportion of involuntary sex than males.

**Table 2:** Individual behavior among sexually-active adolescents in Bangkok, 2020 (n = 872)

Variables	Categories	Total (n = 872)		Sex				P-Value
				Male (n = 367)		Female (n = 505)		
		n	%	n	%	n	%	
Watch porn media	No	269	30.8	53	14.4	216	42.8	< 0.001
	Yes	603	69.2	314	85.6	289	57.2	
Was physically or emotionally abused	No	675	77.4	291	79.3	384	76.0	0.257
	Yes	197	22.6	76	20.7	121	24.0	
Was sexually abused	No	753	86.4	313	85.3	440	87.1	0.434
	Yes	119	13.6	54	14.7	65	12.9	
Experienced physical or emotional neglect	No	674	77.3	291	79.3	383	75.8	0.230
	Yes	198	22.7	76	20.7	122	24.2	
Gambles	No	587	67.3	224	61.0	363	71.9	0.001**
	Yes	285	32.7	143	39.0	142	28.1	
Drinks alcohol	No	515	59.1	236	64.3	279	55.2	0.007*
	Yes	357	40.9	131	35.7	226	44.8	
Smokes cigarettes	No	685	78.6	260	70.8	425	84.2	< 0.001
	Yes	187	21.4	107	29.2	80	15.8	
Uses cannabis	No	812	93.1	330	89.9	482	95.4	0.001**
	Yes	60	6.9	37	10.1	23	4.6	
Use e-cigarettes	No	691	79.2	275	74.9	416	82.4	0.007*
	Yes	181	20.8	92	25.1	89	17.6	
Had an unintended pregnancy	No	776	89.0	343	93.5	433	85.7	< 0.001
	Yes	96	11.0	24	6.5	72	14.3	
Uses birth control	No	272	31.2	136	37.1	136	26.9	0.001**
	Yes	600	68.8	231	62.9	369	73.1	
Uses emergency contraception	No	803	92.1	347	94.6	456	90.3	0.022*
	Yes	69	7.9	20	5.4	49	9.7	

*p-value < 0.05, **p-value < 0.001

### Risky sexual behavior (RSB) among study participants

RSB was measured across six indicators. As noted above, 7.8% reported that their first experience of sex was involuntary; 11.4% had coercive sex in the past year; 33.7% reported not using a condom at their sexual debut; 30.5% reported not using a condom the last time they had sex; 42.4% had more than one sex partner; and 3.0% had received cash or in-kind compensation for sex in the past year. Using these criteria, two out of three of the students in this sample (69.5%) had experienced RSB (see Table 3).

**Table 3:** Sexual history and contraceptive use among sexually-active adolescents in Bangkok, 2020 (n = 872)

Variables	Categories	Total (n = 872)		Sex category				P-Value
				Male (n = 367)		Female (n = 505)		
		n	%	n	%	n	%	
First sex was voluntary	Yes	804	92.2	350	95.4	545	89.9	0.003*
	No	68	7.8	17	4.6	51	10.1	
Ever been forced to have sex	No	773	88.6	319	86.9	454	89.9	0.171
	Yes	99	11.4	48	13.1	51	10.1	
Used condoms at first sex	Yes	578	66.3	231	62.9	347	68.7	0.075
	No	294	33.7	136	37.1	158	31.3	
Used condom at last sex	Yes	606	69.5	255	69.5	351	69.5	0.994
	No	266	30.5	112	30.5	154	30.5	
Number of sexual partners in lifetime	One	502	57.6	223	60.8	279	55.2	0.104
	More than one	370	42.4	144	39.2	226	44.8	
Sold sex before	No	846	97.0	351	95.6	495	98.0	0.041*
	Yes	26	3.0	16	4.4	10	2.0	
Engaged in RSB	No	266	30.5	115	31.3	151	29.9	0.650
	Yes	606	69.5	252	68.7	354	70.1	
Used birth control	No	272	31.2	136	37.1	136	26.9	0.001**
	Yes	600	68.8	231	62.9	369	73.1	
Used emergency contraception at last sex	No	803	92.1	347	94.6	456	90.3	0.022*
	Yes	69	7.9	20	5.4	49	9.7	

RBS; risky sexual behavior

*p-value < 0.05, **p-value < 0.001

### Results of multivariable logistic regression analysis

The multivariate logistic regression analysis identified factors that were significantly associated with RSB, as follows: cannabis use in the past year (AOR = 2.84; 95% CI 1.23–6.56); smoking cigarettes in the past 30 days (AOR = 1.79; 95% CI 1.12–2.88); experiencing substantial loss of property from gambling (AOR = 1.81; 95% CI 1.28–2.55); not using contraception (AOR = 2.74; 95% CI 2.91–3.93); and/or having a history of ACEs (AOR = 1.60; 95% CI 1.03–2.56) (see Table 4).

**Table 4:** Factors associated with risky sexual behavior (RSB) among sexually-active adolescents in Bangkok, 2020 (n = 872)

Variables	Categories	RSB n (%)		COR	(95% CI)	AOR	(95%CI)
		No (n = 266)	Yes (n = 606)				
Uses cannabis	No	259 (31.9)	553 (68.1)	1			
	Yes	7 (11.7)	53 (88.3)	3.55**	1.59–7.91	2.84*	1.23–6.56
Drinks alcohol	No	169 (32.8)	346 (67.2)	1			
	Yes	97 (27.2)	260 (72.8)	1.34	0.97–1.76	1.09	0.79–1.51
Smokes cigarettes	No	233 (34.0)	452 (66.0)	1			
	Yes	33 (17.6)	154 (82.4)	2.41**	2.41–3.62	1.79*	1.12–2.88
Uses e-cigarettes	No	225 (32.6)	466 (67.4)	1			
	Yes	41 (22.7)	140 (77.3)	1.65*	1.12–2.42	1.08	0.69–1.68
Gambles	No	205 (34.9)	382 (65.1)	1			
	Yes	61 (21.4)	224 (78.6)	1.55*	1.40–2.67	1.81*	1.28–2.55
Uses birth control	Yes	218 (36.3)	382 (63.7)	1			
	No	48 (17.6)	224 (82.4)	2.66**	1.87–3.79	2.74**	2.91–3.93
Was sexually abused*	No	238 (31.6)	515 (68.4)	1			
	Yes	28 (23.5)	91 (76.5)	1.65	1.06–2.59	1.60*	1.03–2.56

*p-value < 0.05, **p-value < 0.001. Controlling for socio-demographic characteristics

## Discussion

This study assessed RSB and related factors among sexually-active adolescents enrolled in high school Years 2 and 5, and 2nd year of vocational school, in Bangkok. The students ranged in age from 14–16 years. Substance use variables, such as drinking alcohol, consuming marijuana, and smoking e-cigarettes had a significant association with RSB. Other correlates of RSB include gambling, having had an accidental pregnancy, not using birth control when having sex, and had a history of ACEs. In this study, 14.1% (95% CI 13.3, 15.0) of all students in the participating schools had ever had sex before. A study of sexual behavior among vocational students in northern Thailand found that 46.5% had ever had sex, and males were more sexually active than their female counterparts [7]. This difference with the Bangkok study may be due to varying socio-demographic and cultural contexts between the two study areas. A study in South Korea [23], found that only 5% of high school students were sexually active, which is in stark contrast to a study in the Poland where more than 78% of students attending universities had ever had sex [24]. It is not clear whether these wide differences in sexual activity are attributed to different definitions of sex, difference in age of the samples, or other artifacts of the studies.

### The prevalence of risky sex behavior (RSB)

Most studies have defined RSB as (1) Not using condoms for every episode of sex; and (2) Having two or more sexual partners. In adolescents, RSB includes sexual debut at a very young age, a factor which is also associated with reproductive health risk, such as unprotected sex. Use of mood-altering drugs before or during sex also contributes to RSB [25]. Some studies define RSB differently as, for example: (1) Number of sexual partners during the lifetime; (2) Inconsistent use of condoms; and (3) Unwanted or unplanned pre-marital pregnancy [26, 27]. Others also refer to exchanging sex for cash or in-kind compensation as RSB [28]. RSB can produce negative consequences beyond the individual, such as family conflict, damage to relationships, legal disputes, and/or financial problems [29]. Unprotected sex is often the focus as a root cause of these problems because of its association with STIs and unwanted pregnancy. Worldwide, unprotected sex for both male and female adolescents ranks second in the health risk category [30]. Over the past 15 years, a large proportion of adolescents in Central and Eastern Europe reported having RSB, which is associated with an increase in STIs other than HIV. The same is true for teen pregnancy and abortion rates [31]. These public health issues have prompted research into the factors influencing adolescent RSB [32]. In addition, definitions of RSB are becoming more standardized, and they now include coercion at sexual debut and forced sex in the past year as criteria. These factors have clinical utility and can be integrated into RSB prevention education programs.

The prevalence of RSB among college and university students in Ethiopia ranged from 23.3% to 60.9%. The estimated prevalence of RSB among college and university students was 40.6% and 42.4%, respectively, and the estimated overall prevalence of RSB was 41.6% [13]. Those results reflect a higher level of RSB than found in other studies conducted in Africa, North America, and Europe. Differences may be due to sample size, age of the sample, calculation of the rates, and definition of RSB [26].

### Factors associated with RSB

Adolescent RSB represents a serious public health concern that is potentially detrimental to health and well-being. RSB (e.g., multiple sex partners, not using condoms) may negatively affect health by directly contributing to risk for STIs and unintended pregnancy [25]. There is significant evidence suggesting that having a history of child sexual abuse contributes to RSB in adolescents [26]. Adolescents who were sexually abused at a young age are at higher risk of premature sex, having sex with multiple partners, having sex under the influence of drugs, having sex with an uncommitted partner, and having unprotected sex [33]. Consistent with this study, adolescents exposed to sexual abuse had 1.7 times more chance of experiencing RSB than adolescents without a history of sexual abuse.

Results reveal a high prevalence of adolescent contraceptive use in Bangkok. More than two-thirds of sexually-active teens in this study have used birth control. Condoms are the most common method of birth control for this sample. This is consistent with previous evidence highlighting condoms as the most common method of contraception among adolescents [7, 34], followed by emergency contraception, and withdrawal [35]. This study did not examine factors which might influence a teen’s decision to use contraception. However, the findings of some studies suggest that the preference for condoms stems from adolescent concern about perceived side effects of other methods [36]. Adolescent females report using contraception more than their male counterparts. This is different from previous studies in Africa and Asia that found that adolescent males were more likely to use contraception with their female partners [37]. The differences may be attributable to the phrasing of questions about using contraception, cultural differences across countries, and knowledge acquisition through online sources.

The relationship between adolescent RSB and substance use has been analyzed. There is evidence from studies that adolescent substance use is predictive of later RSB. Sexually-active teenagers may begin to spend more time with other sexually-active friends, and find themselves in social environments that facilitate substance abuse [11, 38]. Adolescent RSB is more common when use of mood-altering substances is involved [39]. A study found that use of addictive drugs before last sex was prevalent among adolescents [38]. This study provides some evidence that is consistent with a possible explanation for the relationship between RSB and adolescent substance use. Substance use disorders are often associated with RSB. Our findings are inconsistent with recent studies reporting that alcohol use is more likely to lead to unprotected sex [12–15]. The association between alcohol use and RSB is empirically supported in a variety of studies. However, most of those studies are cross-sectional and, thus, causality cannot be inferred. The relationship between alcohol use and RSB is complex, and may be influenced by a combination of social, physiological, and personality traits [12].

Adolescent cannabis use is a growing public health problem. In this study, the overall prevalence of cannabis use in the past year was approximately 6.9%, and adolescents who used cannabis had 3.7 times more chance of experiencing RSB than adolescents who did not use cannabis. These findings are consistent with previous research which found an association between marijuana use and an increased risk of sex among a sample of adolescents. [39]. Marijuana use is prevalent in high-income countries. For example, in the United States, approximately 0.7% of 13–14-year-olds and 3.4% of 15–16-year-olds use marijuana daily, and 22.2% of teens report using marijuana in the past month [40]. The use of marijuana may affect RSB. For example, studies have found that marijuana users are more likely to have their first sex at an earlier age than non-users. Plus, marijuana has been linked to RSB, such as having multiple sex partners. It has been speculated that cannabis use may increase the risk of RSB through negative effects on neurodevelopmental or decision-making abilities [19]. Other factors include personality traits, self-perception, social status, and attractiveness. The main risks from marijuana use include premature sex (e.g., having sex before age 15), promiscuity, and the accompanying risk of STIs, HIV, and unplanned pregnancy [19, 41].

This study found that one out of three sexually-experienced adolescents had considerable loss of property from gambling. Adolescents who gamble had 1.7 times more chance of RSB compared to adolescents who did not gamble. Male adolescents had more gambling behavior than their female counterparts. This contrasts with some previous studies which found variable prevalence of gambling among adolescents, with 77% to 88% of adolescents and young adults generally engaging in one form of gambling or another [42]. That prevalence rate is relatively high compared to the older population, with a prevalence of gambling between 39 and 50% [43]. Adolescents are more likely to start social gambling with friends and family, and gambling has the potential to negatively affect an individual's well-being, which may include issues related to relationships, family, finances, social status, and career pursuits [44]. According to a study in Uganda, 62% of youth gambled, and gamblers were more male than female. The findings from the current study also found that male adolescents gamble more than female adolescents [45]. A systematic review of national studies published in 2014 reported that the prevalence of problem gambling in North America was in the range of 2.1% to 2.6%, and in Oceania from 0.2% to 4.4%. By contrast, in Europe, the prevalence of problem gambling ranged between 0.2% and 12.3%. These results are hard to verify and may differ across countries and settings due to response bias and survey methodology. Additionally, studies may differ significantly based on inclusion criteria and target populations. These methodological discrepancies have led to outcomes that vary greatly from country to country or between different studies in the same country [45]. The vast majority of adolescents who gamble consider themselves prone to problem behaviors, and they may not see a link between gambling and health risk, particularly RSB. Gambling is associated with emotional, physical and mental problems, and many adolescents who gamble excessively experience stress, anxiety, and depression. Gambling addicts often experience mood swings that reflect their winnings and losses, and makes them prone to sudden anger and irrational outbursts. These mood disorders also make gamblers more vulnerable to unprotected sex and sex with multiple partners, or even exchanging sex for cash or in-kind compensation if gambling debt becomes too onerous [45].

Although the study reflects some acceptable quality assurance outcomes, the RSB data in this study were self-reported and, thus, subjects may under- or over-report their own RSB. Also, the survey focused on RSB in the past year, and targeted adolescents in high school Years 2 and 5, and 2nd year vocational school students. Thus, the sample is probably not representative of all adolescents, either in Bangkok or Thailand generally. Researchers need to consider studies using community-based qualitative research methods to determine strong predictors of RSB among adolescents. Inquiries about sexual harassment may have inappropriate metrics such as excluding the severity, duration, and frequency of the harassing behavior, and how that affects RSB.

## Conclusions

This study assessed RSB and related factors among sexually-active adolescents enrolled in high school Years 2 and 5, and 2nd year vocational schools in Bangkok. The students ranged in age from 14 to 16 years. About 14% of the study participants had engaged in sexual intercourse and, of these more than two-thirds can be considered to have RSB. The findings of this study show that substance use variables, such as consuming marijuana and smoking cigarettes had a significant association with RSB. Other correlates of RSB include gambling, not using birth control during sex, and having a history of ACEs. This study found that the majority of sexually-active adolescents are engaging in RSB. Interventions at the health facility, community, and school level should focus on the identified determinants of RSB among adolescents to minimize adverse consequences.

## Data Availability

The data set gathered and/or analyzed for the current study will be made accessible upon request of the corresponding author.

## Abbreviations

RSB: Risky sexual behavior
STIs: Sexually transmitted infections
ACEs: Adverse childhood experiences
